# Comparative genomic analysis of head and body/tail of pancreatic ductal adenocarcinoma at early and late stages

**DOI:** 10.1111/jcmm.16281

**Published:** 2021-01-16

**Authors:** Xueyou Zhang, Shi Feng, Qian Wang, Haitao Huang, Ruihan Chen, Qinfen Xie, Wu Zhang, Aodi Wang, Shuirong Zhang, Lingjian Wang, Ming Yao, Qi Ling

**Affiliations:** ^1^ Department of Surgery The First Affiliated Hospital Zhejiang University School of Medicine Hangzhou China; ^2^ Department of Pathology The First Affiliated Hospital Zhejiang University School of Medicine Hangzhou China; ^3^ OrigiMed, Inc. Hangzhou China; ^4^ Department of Surgery Shulan (Hangzhou) Hospital Hangzhou China

**Keywords:** body/tail, druggable, genomic profiling, head, pancreatic ductal adenocarcinoma, tumour location

## Abstract

Pancreatic ductal adenocarcinoma (PDAC), one of the most lethal human cancers, can be divided into head and body/tail cancers anatomically. We previously reported a prognostic relevance of tumour location in resectable PDAC. This study aimed to further explore the mechanism underlying the molecular diversity between the head and body/tail of PDACs. We detected tumour genomes in 154 resectable (surgery) and non‐resectable (biopsy) PDACs using a next‐generation sequencing panel. Wilcoxon's rank test or Fisher's exact test was used for evaluating associations between clinical characteristics, mutation frequency and survival probability between the two cohorts. Compared with pancreatic head cancers, pancreatic body/tail cancers showed significantly more enriched genomic alterations in *KRAS* (97.1% vs 82.4%, *P* = 0.004) and *SMAD4* (42.0% vs 21.2%, *P* = 0.008). At early stages (I‐II), the *SMAD4* mutation rate was significantly higher in pancreatic body/tail cancers than pancreatic head cancers (56.0% vs 26.5%, *P* = 0.021). At late stages (III‐IV), pancreatic body/tail cancers presented significantly higher *KRAS* mutation rate (100.0% vs 75.8%, *P* = 0.001), higher frequency of MAPK pathway mutation (100% vs 87.8%, *P* = 0.040) and lower rates of druggable genomic alterations (30.8% vs 57.6%, *P* = 0.030) than pancreatic head cancers. Our work points out that pancreatic body/tail cancer seems to be more malignant than pancreatic head cancer at late stages.

## 1 INTRODUCTION

Pancreatic ductal adenocarcinoma (PDAC), one of the most lethal human cancers, can be divided into head and body/tail cancers according to the anatomy. We previously made a comprehensive review on the diversity between pancreatic head and body/tail cancers in tissue ontogeny (eg cell composition, blood supply, lymphatic and venous backflow), clinical parameters (eg presentation, treatment and prognosis) and in vitro genetic and tumour biology.^1^ We assumed that pancreatic body/tail cancer might be a less malignant phenotype compared with pancreatic head cancer. The conclusion was further supported by a recent large cohort database analysis which indicated that patients with pancreatic body/tail cancers had a better prognosis compared to those with head cancers among resectable PDACs.^2^

Because of the genomic heterogeneity of PDACs, identification of the genetic and epigenetic profile of pancreatic head and body/tail cancers could be of great help to better understand the prognostic relevance of primary tumour location. We previously demonstrated that pancreatic body/tail cancer had less invasiveness and metastasis potential than pancreatic head cancer possibly via miR‐501‐3p/E‐cadherin signalling by using strictly matched resectable pancreatic head and body/tail cancers in both in vitro and in vivo models.^3^ In contrast, Birnbaum et al^4^ and Dreyer et al^5^ identified genomic and transcriptional diversities between the two subtypes of resectable PDACs and drew an opposite conclusion that pancreatic body/tail cancer had aggressive tumour biology and worse clinical outcome. The shortcoming of these previous studies was that almost all of the tissue samples were obtained from an early‐stage (I‐II) resectable PDACs. To further clarify different genomic signatures between pancreatic head and body/tail cancers, we included PDAC samples from both early stage and late stage and analysed in‐depth molecular characterization in an independent Chinese PDAC cohort by targeted next‐generation sequencing (NGS). Our research might provide another picture of the molecular aspect for a better understanding of PDACs, and the potential strategies for targeted therapy for patients with PDACs.

## 2 MATERIALS AND METHODS

### 2.1 Patients

Tumour and matched peripheral blood samples from 154 patients with treatment‐naive PDACs, including 85 pancreatic head cancers and 69 pancreatic body/tail cancers, were involved in the research. The median follow‐up time was 1.6 (range: 0.2‐3.9) years. Surgery and biopsy tumour samples were available in 102 (63 head and 39 body/tail cancers) and 52 (22 head and 30 body/tail cancers) patients, respectively. A total of 103 and 51 patients were collected from the First Affiliated Hospital of Zhejiang University School of Medicine and Shulan (Hangzhou) Hospital, respectively. The study was approved by the Ethics Committee of the First Affiliated Hospital, Zhejiang University School of Medicine and the Ethics Committee of Shulan Hospital and followed the Declaration of Helsinki. All patients had signed the informed consent.

### 2.2 Sequencing experiment

A total of 154 specimens and matched normal blood were detected and analysed in a College of American Pathologists (CAP) and Clinical Laboratory Improvement Amendments (CLIA) certified laboratory at OrigiMed for CSYS assay^6^ with a mean coverage of 900× for tumour samples (minimum 700×) and 300× for matched normal blood samples. This panel covers all the coding exons of 450 cancer‐related genes and 64 selected introns of 39 genes that frequently rearranged in solid tumours (Table S1). We analysed multiple genomic variant types, including single nucleotide variants (SNVs), copy number variations (CNVs), short and long insertions/deletions (indels) and gene rearrangements by bioinformatics workflows described previously.^6^ White blood cells isolated from whole blood were used as matched normal control to identify somatic genomic alterations from germline mutations.

### 2.3 Statistics analysis

Wilcoxon's rank test or Fisher's exact test was used for evaluating associations between clinical characteristics, mutation frequency and survival probability between the two cohorts. *P* < 0.05 was considered statistically significant. Statistical analysis was performed using R 3.3.1.

## 3 RESULTS

### 3.1 Patient characteristics and overall survival

**FIGURE 1:**
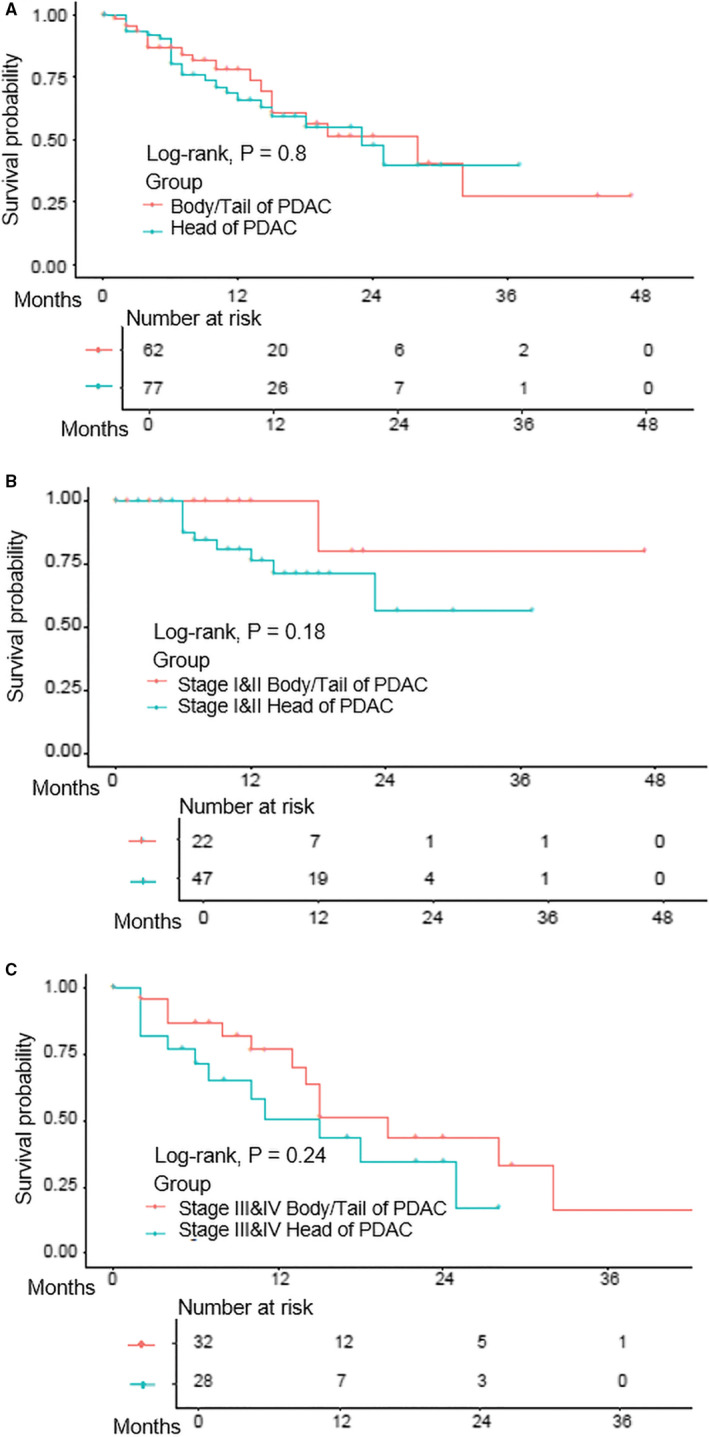
The comparison of patient survival between pancreatic head and body/tail cancers. A, The comparison of overall survival in all PDACs; B, the comparison of overall survival in early (I‐II) pancreatic head and body/tail cancer; and C, the comparison of overall survival in the late‐stage (III‐IV) pancreatic head and body/tail cancer. PDAC indicates pancreatic ductal adenocarcinoma

The patient clinicopathological data are summarized in Table 1. At the initial diagnosis, compared with pancreatic head cancers, pancreatic body/tail cancers showed more advanced stage (III‐IV) and distant metastasis, but less local invasion. There is no significant difference in overall patient survival between the two subgroups (Figure 1A). The median survival time was 23 and 28 months for patients with pancreatic head cancers and pancreatic body/tail cancers respectively with no statistic difference. Also, there was no significant difference in survival for tumours at early stages (I‐II) (*P* = 0.180, Figure 1B) and late stages (III‐IV) (*P* = 0.240, Figure 1C).

**TABLE 1:** Overview of patients’ clinicopathological characteristics

Characteristic	Head of PDAC (n = 85)	Body/tail of PDAC (n = 69)	*P*
Age (y)	61 (38‐86)	62 (42‐78)	.540
Male, n (%)	55 (64.7)	45 (65.2)	1.000
Tumour stage, n (%)			
I	21 (24.7)	13 (17.4)	.040
II	28 (32.9)	13 (17.4)	
III	10 (11.8)	6 (8.7)	
IV	23 (27.1)	33 (47.8)	
Undefined	3 (3.5)	4 (5.8)	
Tumour grade, n (%)			
High	1 (1.2)	1 (1.4)	.845
Moderate	15 (17.6)	12 (17.4)	
Low	32 (37.6)	20 (30.0)	
Undefined	37 (43.5)	36 (52.2)	
Metastasis, n (%)			
Lymph node	25 (54.3)	16 (53.3)	1.000
Liver	12 (21.4)	17 (39.5)	.074
Other	3 (5.5)	8 (19.5)	.050
Local invasion, n (%)	52 (96.3)	35 (83.3)	.039

### 3.2 The landscape of genomic alterations

There were 216 genes found to be mutated in PDAC samples, with a mutation rate ranging from 0.6% to 89.0%. Thirteen genes showed mutation frequencies over 5%, and the TOP 5 most frequently mutated genes were *KRAS* (89.0%), *TP53* (81.8%), *SMAD4* (30.5%), *CDKN2A* (29.9%) and *ARID1A* (16.2%).

**FIGURE 2:**
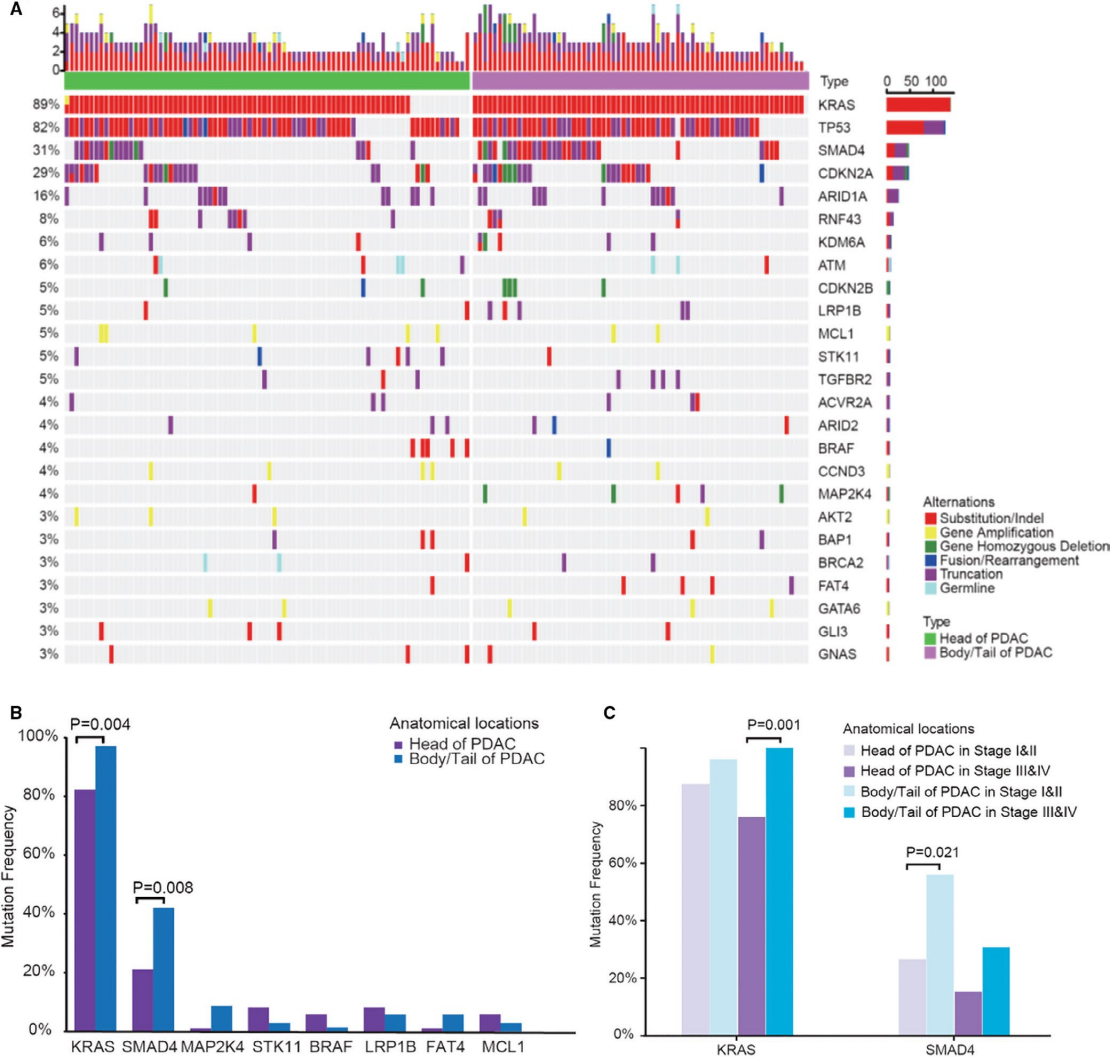
The comparison of genomic alterations between pancreatic head and body/tail cancers. A, The genomic landscape in head (n = 85) and body/tail (n = 69) of PDACs; B, eight gene alteration frequencies in head and body/tail of PDACs. The comparison of overall survival in all PDACs; C, *KRAS* and *SMAD4* alteration frequencies in head and body/tail of PDACs related to early (I‐II) and advanced (III‐IV) stages. PDAC indicates pancreatic ductal adenocarcinoma

The comparison of gene alteration frequencies between pancreatic head and body/tail cancers is shown in Figure 2A. Compared with pancreatic head cancers, pancreatic body/tail cancers showed significant higher clinically relevant mutation frequency in *KRAS* (97.1% vs 82.4%, *P* = 0.004) and *SMAD4* (42.0% vs 21.3%, *P* = 0.008) (Figure 2B). The mutation frequencies of these two genes were further analysed according to tumour stage (Figure 2C). The statistical significance in *KRAS* and *SMAD4* mutation rates was achieved only at late stages and early stages, respectively. Interestingly, all *MAP2K4* mutations (n = 6) occurred in advanced PDACs.

The comparison of hotspot mutation sites is shown in Figure S1. Codon 12 and codon 13 were the major hotspots of *KRAS* in solid tumours. In this study, G12V/D/R contributed 87.7% of *KRAS* mutations. However, there was no significant difference in the detail composition of G12V/D/R between the pancreatic head and body/tail cancers.

### 3.3 Pathway analysis

**FIGURE 3:**
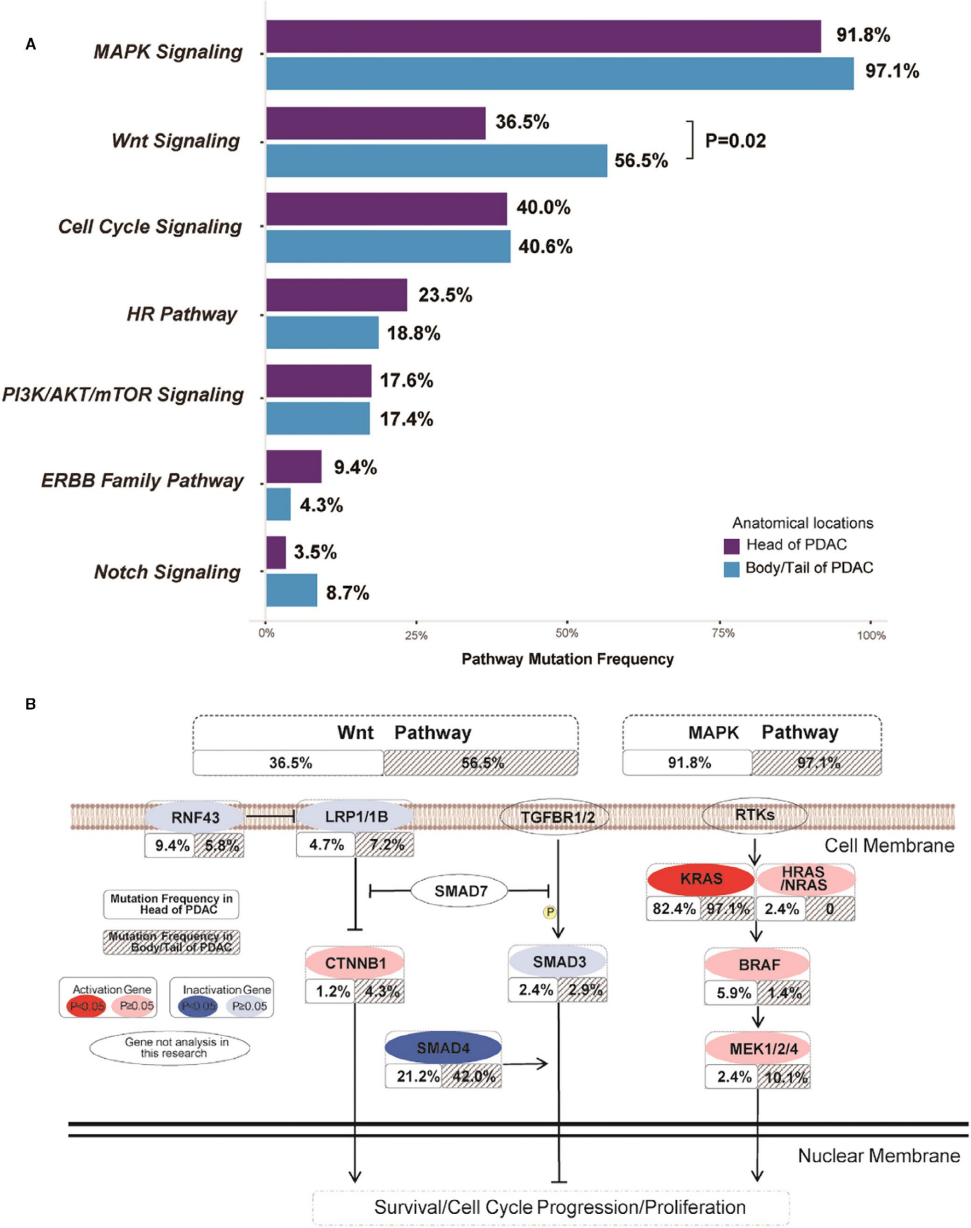
The comparison of mutation frequencies in pathways. A, Seven gene pathways mutation frequencies in head and body/tail PDAC; B, TOP 2 mutated pathways in gene level

Seven primarily signalling pathways influencing tumour initiation and progression were involved in the pathway comparison analysis, including MAPK signalling, Wnt signalling, cell cycle signalling, homologous recombination (HR) pathway, PI3K‐AKT‐mTOR signalling, ERBB family pathway and Notch signalling. Among these pathways, the total mutation frequency of genes involved in Wnt pathway was significantly higher in pancreatic body/tail cancers than pancreatic head cancers (56.5% vs 36.5%, *P* = 0.020) (Figure 3A). We further investigated whether this difference in mutation frequency in Wnt pathway genes related to tumour stages. The mutation frequency of the Wnt pathway genes did not show statistical difference but a higher trend both in early stage (65.4% vs 42.9%, *P* = 0.090) and in late stage (48.7% vs 30.3%, *P* = 0.150) between pancreatic body/tail and head cancers. MAPK pathway was the most dominant mutated pathway both in pancreatic head and in body/tail cancers (91.8% vs 97.1%, *P* = 0.188), although there was no significant difference. Of note, the mutation frequency of MAPK pathway showed the apparent difference in advanced pancreatic body/tail and head cancers (100% vs 87.8%, *P* = 0.040), but not in early‐stage cancers (92.3% vs 95.9%, *P* = 0.600).

Besides, we focused on TOP 2 frequently mutated pathways, Wnt and MAPK signalling at the gene levels (Figure 3B). Higher mutated frequency in Wnt signalling of pancreatic body/tail cancers mainly resulted from distinctly enriched *SMAD4* mutations (*P* = 0.008), and more abundant *LRP1/1B* and *CTNNB1* mutations. Interestingly, we found that *RNF43* mutations (n = 8) were mutually exclusive to *SMAD4* in pancreatic head cancers but 3 of 4 *RNF43* mutations co‐occurred with *SMAD4* in pancreatic body/tail cancers.

### 3.4 Clinical druggable genes

**FIGURE 4:**
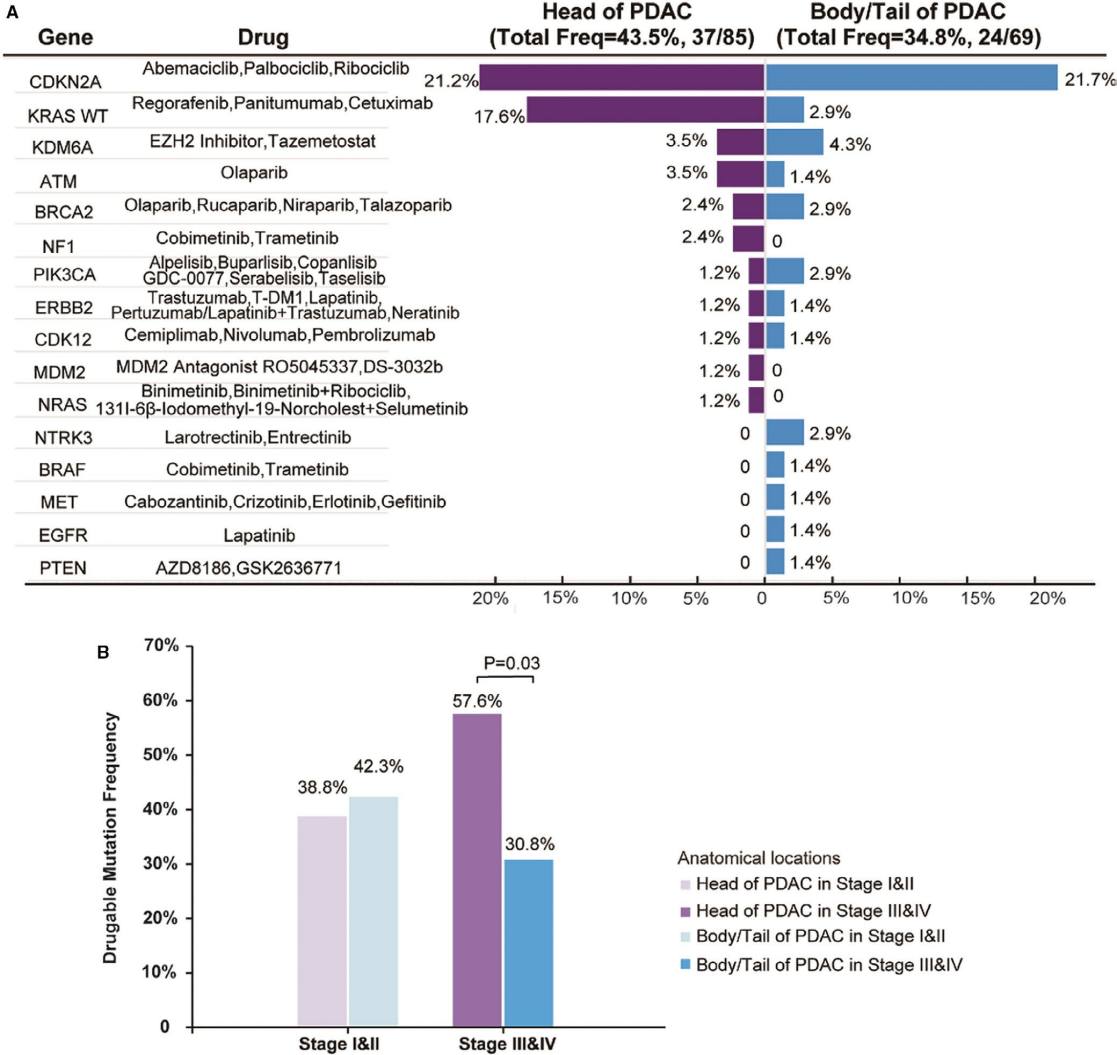
The comparison of putative druggable genome in head and body/tail PDAC (A) and druggable mutation frequency in head and body/tail of PDAC according to early and advanced stages (B). PDAC indicates pancreatic ductal adenocarcinoma

To compare potential clinical benefits among pancreatic head and body/tail cancers in terms of targeted drugs, we analysed actionable alterations from 16 clinical relevant genes with 43 potential therapies according to the widely accepted rule (Figure 4A).^7^ Overall, 43.5% of pancreatic head cancer and 34.8% of pancreatic body/tail cancer carried at least one genomic alteration that could potentially benefit from the targeted drugs, but no significant difference was achieved. In late stages (III‐IV), pancreatic body/tail cancer showed significant less druggable mutation than pancreatic head cancer (30.8% vs 57.6%, *P* = 0.030) (Figure 4B).

The TOP 3 frequently mutated targets were *CDKN2A* (21.2%), *KRAS* wild‐type (17.6%) and *KDM6A*/*ATM* (3.5%) in pancreatic head cancers and *CDKN2A* (21.7%), *KDM6A* (4.3%) and *KRAS* wild‐type/*BRCA2*/*PIK3CA*/*NTRK3* (2.9%) in pancreatic body/tail cancers. There was significantly less *KRAS* wild‐type in pancreatic body/tail cancers than pancreatic head cancers (2.9% vs 17.6%, *P* = 0.004). In late stages (III‐IV), the difference in *KRAS* wild‐type druggable mutation frequency between the two subtypes was increased (0% vs 24.2%, *P* = 0.001). In addition, patients with TMB‐H (>10 mutations/Mb) accounted for 4.7% (n = 4) and 1.4% (n = 1), in pancreatic body/tail and head cancers, respectively (*P* = 0.381).

## 4 DISCUSSION

It is well known that patients with pancreatic body/tail cancer usually have a poorer prognosis than those with pancreatic head cancer probably because of more advanced pathologic stages at initial diagnosis.^1^ To better understand the prognostic relevance and distinct tumour biology, we should compare the two subtypes of PDACs in comparable conditions such as AJCC TNM stages and pathology grade. A large cohort analysis using the National Cancer Database of the United States from 1998 to 2011 demonstrated that among 40,980 cases of resected PDAC, pancreatic head cancers had advanced tumour stage, higher nodal positivity, worse tumour grade and poorer overall survival than pancreatic body/tail cancers.^2^ A recent strictly propensity score‐matched (eg race, gender, marital status, TMN stage and pathology grade) study including 4,571 resected T1 stage PDACs from Surveillance, Epidemiology, and End Results (SEER) database (2004‐2014) provided the best evidence showing the prognostic value of tumour location in early‐stage PDACs up to now.^8^ They found that patients with pancreatic head cancer had a worse prognosis compared to those with pancreatic body/tail cancers. Body/tail location was further proved to be an independent indicator for better chances of survival in T1 PDAC patients. In this sense, pancreatic body/tail cancer seems to be a less malignant phenotype as compared to pancreatic head cancer.

**FIGURE 5:**
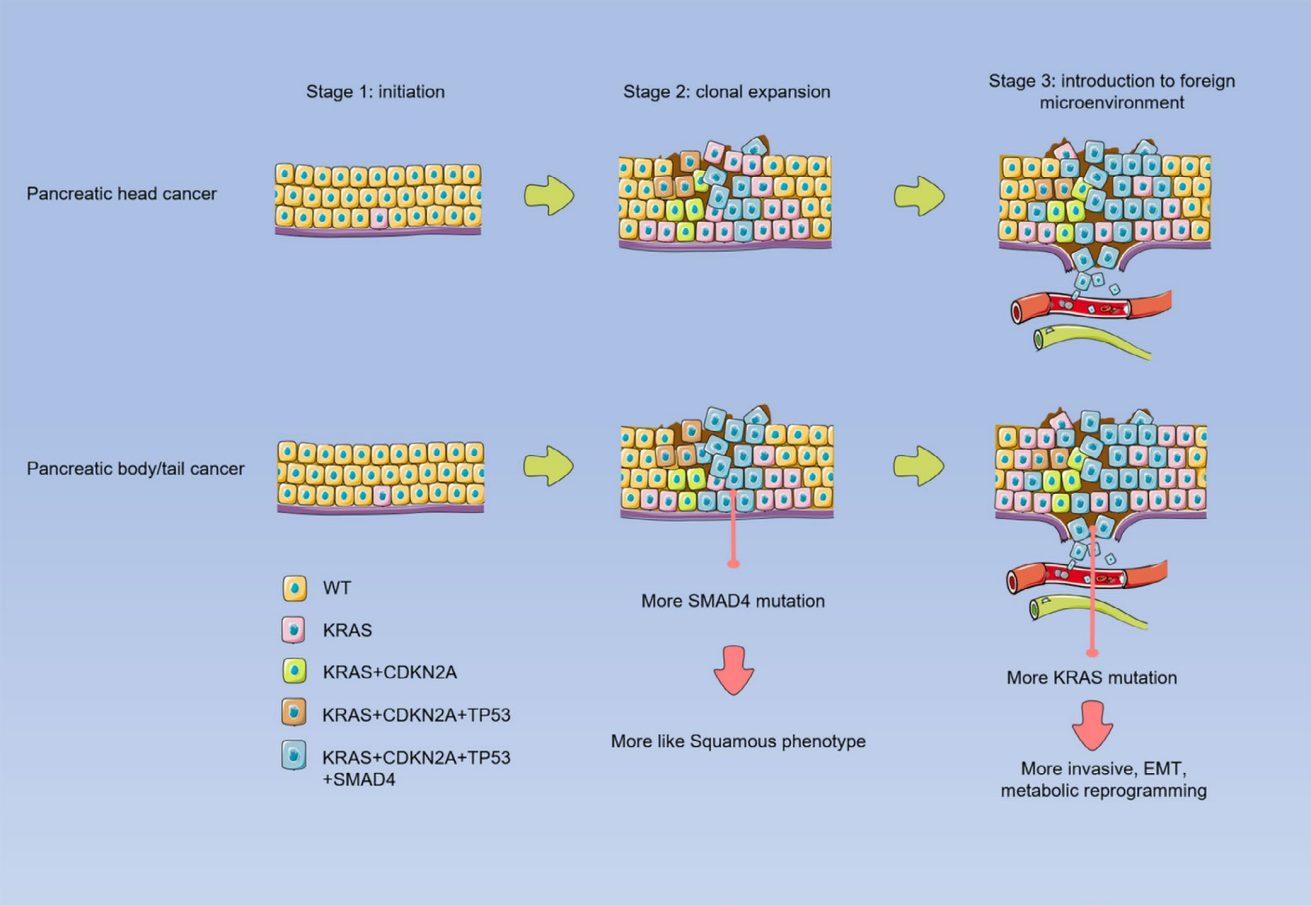
Schematic illustration showing the molecular diversity between pancreatic head and body/tail cancers. The tumour staging (stage 1, tumour initiation; stage 2, the clonal expansion phase; stage 3, interaction with microenvironment) was defined according to Makohon‐Moore's study^10^

However, the genomic and transcriptomic profiling comparison between pancreatic head and body/tail cancers using resected PDAC samples provided the opposite results. Both Birnaum's^4^ and Dreyer's^5^ studies showed pancreatic body/tail cancers were associated with ‘squamous phenotype’^9^ and presented more aggressive tumour biology such as epithelial‐to‐mesenchymal transition (EMT), inflammation and metabolic reprogramming. This study displayed a twofold higher *SMAD4* mutation rate in pancreatic body/tail cancers than pancreatic head cancers among early‐stage tumours (I‐II). Inactivating mutation in *SMAD4* does not initiate the tumour genes in PDAC but serve as secondary genetic alterations following *KRAS* mutation,^10^ which was consistent with our genomic finding that both *KRAS* and *SMAD4* had higher mutation frequency in pancreatic body/tail cancers. Interestingly, SMAD4/TGF‐beta signalling is involved in squamous/EMT transition in PDAC.^11^ Therefore, our results supported the previous two studies that pancreatic body/tail cancers might be more ‘squamous phenotype’ than pancreatic head cancers. We also assume that the molecular diversity between pancreatic head and body/tail cancers starts from the clonal expansion phase^10^ but not the tumour initiation (Figure 5).

When PDAC developed to the late stages (III‐IV), there was a remarkable difference in cancer genome between the two subtypes. We found significantly higher *KRAS* mutations (eg substitution, indels and gene amplification), more enriched mutation frequencies in MAPK pathway and a lower rate of druggable genomic alterations in pancreatic body/tail cancers than pancreatic head cancers. Activating mutation of *KRAS* is almost ubiquitous (~95%) in PDAC and is an essential event in both tumour initiation and progression.^12^ Mutant *KRAS* drives PDAC development and promotes tumour cell proliferation via altered metabolic pathways including stimulation of glucose uptake and utilization, reprogrammed glutamine metabolism and increased autophagy.^12^ Furthermore, *KRAS* driver mutations lead to the activation of Wnt and MAPK pathways, which controls tumour cell proliferation, motility, metabolism and survival.^13^ Silencing of mutant *KRAS* by CRISPR‐CasRx system suppresses PDAC progression.^14^ The results were consistent with a large cohort study including 9,952 patients with metastasized PDAC from the Netherlands Cancer Registry (2005‐2015), which demonstrated that pancreatic tail cancers had more metastatic sites and worse survival.^15^ It seems that pancreatic body/tail cancers are more biological aggressive than pancreatic head cancers as PDAC has progressed to late stage.

Besides, the genomic variant results indicated that the choice of therapy strategies could refer to tumour location and mutated genes. Birnbaum et al^4^ suggested checkpoint inhibitors as neoadjuvant treatment for pancreatic head cancers and anti‐EGFR targeted therapies for pancreatic body/tail cancers. This study displayed a significantly lower rate of *KRAS* wild‐type in pancreatic body/tail cancers than pancreatic head cancers, indicating a low efficiency of regorafenib, panitumumab and cetuximab, which are FDA‐approved drugs for the treatment of colorectal cancer patients with *KRAS* wild‐type. A novel small‐molecular inhibitor AMG510 targeting to a *KRAS* mutation G12C presented valid anti‐cancer ability for patients with non–small‐cell lung cancer in the phase I clinical trial^16^ and may be of help for the 100% *KRAS* mutated late‐stage pancreatic body/tail cancers. Anti‐EGFR or BRAF targeted therapies were not recommended in either pancreatic head or body/tail cancers because of the extremely low druggable mutation frequencies.

There were strengths and limitations in this study. This was the first study including both early‐ and late‐stage PDACs and comparing the genomic profiles between pancreatic head and body/tail cancers. Thus, it is a more comprehensive comparison than the previous studies. But the sample size was rather small. Moreover, this study used ultra‐deep panel sequencing to detect extremely low frequencies. But the panel was limited to 450 genes, and the previously defined molecular subtypes^17^ could not be analysed. We should also note that the intra‐tumoral heterogeneity, which has been described in PDACs,^18^ may influence our results.

## ETHICS APPROVAL AND CONSENT TO PARTICIPATE

The study was approved by the Ethics Committee of the First Affiliated Hospital, Zhejiang University School of Medicine and the Ethics Committee of Shulan Hospital, and followed the declaration of Helsinki. All patients had signed the informed consent.

## CONFLICT OF INTEREST

QW, AW, SZ, LW and MY are employees of OrigiMed.

## AUTHOR CONTRIBUTION

**Xueyou Zhang:** Methodology (lead); Writing‐original draft (lead). **Shi Feng:** Formal analysis (lead). **Qian Wang:** Data curation (lead); Formal analysis (equal). **Haitao Huang:** Formal analysis (equal). **Ruihan Chen:** Formal analysis (equal). **Qinfen Xie:** Investigation (equal). **Zhang Wu:** Investigation (equal). **Aodi Wang:** Data curation (equal). **Shuirong Zhang:** Data curation (equal). **Lingjian Wang:** Data curation (equal). **Ming Yao:** Data curation (equal). **Qi Ling:** Conceptualization (lead); Writing‐review & editing (lead).

## Supporting information

Fig S1

Table S1

## Data Availability

The data sets used and/or analysed during the current study are available from the corresponding author on reasonable request.
