# Supplementary material for: Comparative genomic analysis of head and body/tail of pancreatic ductal adenocarcinoma at early and late stages
Source: J Cell Mol Med. 2021 Jan 16;25(3):1750–8. doi: 10.1111/jcmm.16281 (PMC7875914; doi:10.1111/jcmm.16281)
Supplement: Supplementary file 1 — Fig S1 [file JCMM-25-1750-s001.docx]

**
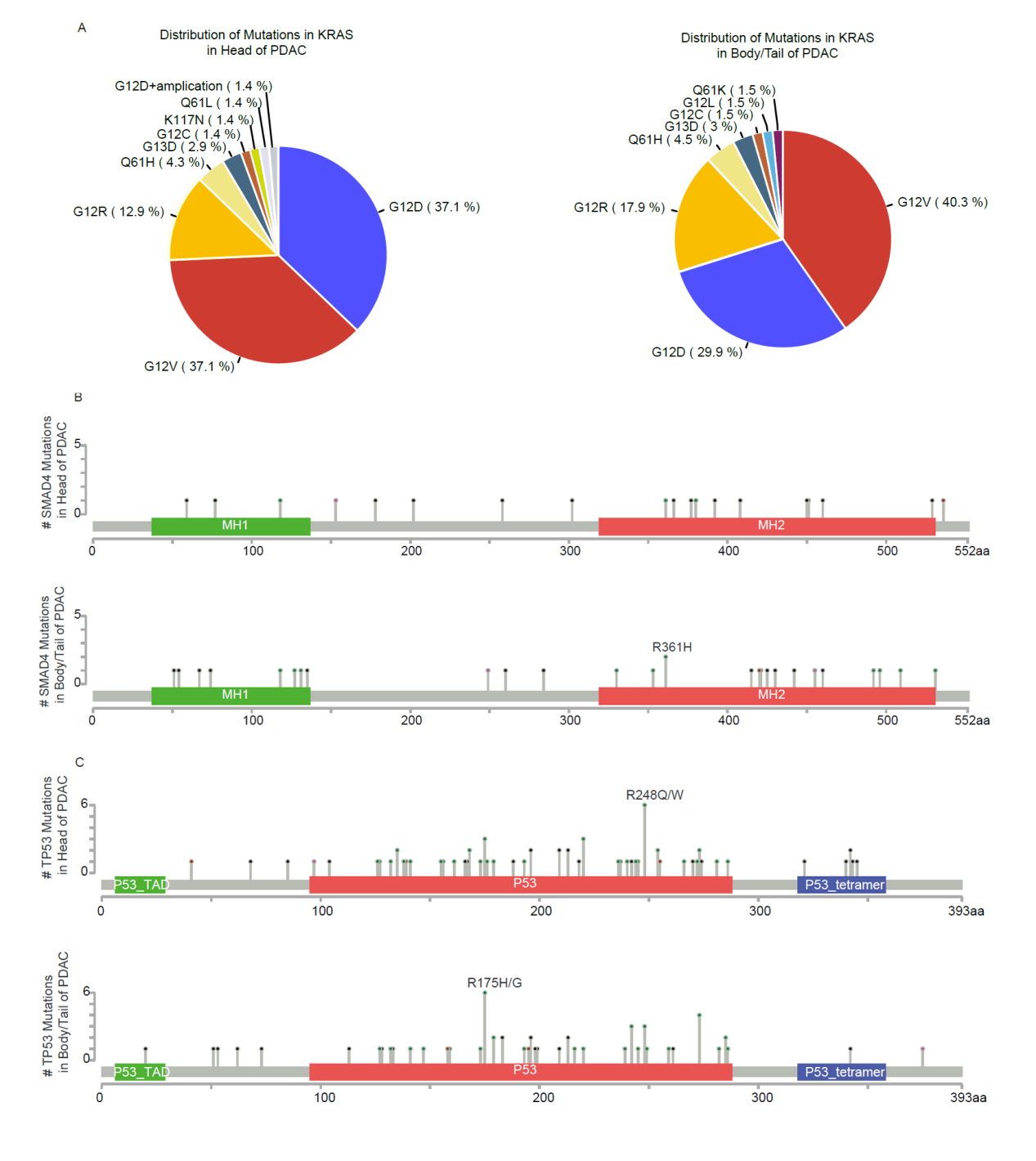
Figure S1.** The comparison of the distribution of somatic mutations in *KRAS, SMAD4* and *TP53* between pancreatic head and body/tail cancers. A. Piechart illustrates the frequency distributions of *KRAS* somatic mutations. B. Lollipop showed the mutation mappers in *SMAD4*. C. Mutation mappers in *TP53* somatic mutations.
